# Characterising innovations in maternal and newborn health based on a common theory of change: lessons from developing and applying a characterisation framework in Nigeria, Ethiopia and India

**DOI:** 10.1136/bmjgh-2019-001405

**Published:** 2019-07-18

**Authors:** Krystyna Makowiecka, Tanya Marchant, Wuleta Betemariam, Anuraag Chaturvedi, Laboni Jana, Audu Liman, Bereket Mathewos, Fatima B Muhammad, Katherine Semrau, Sita Shankar Wunnava, Lynn M Sibley, Della Berhanu, Meenakshi Gautham, Nasir Umar, Neil Spicer, Joanna Schellenberg

**Affiliations:** 1 Department of Infectious Disease Epidemiology, London School of Hygiene and Tropical Medicine, London, UK; 2 Department of Disease Control, London School of Hygiene and Tropical Medicine, London, UK; 3 The Last Ten Kilometers Project, JSI Research and Training Institute, Addis Ababa, Ethiopia; 4 Public Health and Nutrition, Public Health Foundation of India, Gurgaon, Haryana, India; 5 IntraHealth, New Delhi, India; 6 Atiku Centre for Development, American University of Nigeria, Yola, Adamawa, Nigeria (formerly with PACT Nigeria); 7 Save the Children, Addis Ababa, Ethiopia; 8 Society for Family Health, Abuja, Nigeria; 9 Ariadne Labs, Brigham and Women's Hospital, Boston, Massachusetts, USA; 10 Division of Global Health Equity and Department of Medicine, Brigham and Women's Hospital, Boston, Massachusetts, USA; 11 Public Health Consultant, Coimbatore, Tamilnadu, India (formerly with PATH, New Delhi); 12 Emory University, Atlanta, Georgia, USA; 13 Department of Disease Control, London School of Hygiene and Tropical Medicine, London, UK; 14 Department of Global Health and Development, London School of Hygiene and Tropical Medicine, London, UK; 15 Department of Disease Control, London School of Hygiene and Tropical Medicine, London, UK; 16 Department of Global Health and Development, London School of Hygiene and Tropical Medicine, London, UK; 17 Department of Disease Control, London School of Hygiene and Tropical Medicine, London, UK

**Keywords:** ‘theory of change’ maternal, newborn, characterisation

## Abstract

Government leadership is key to enhancing maternal and newborn survival. In low/middle-income countries, donor support is extensive and multiple actors add complexity. For policymakers and others interested in harmonising diverse maternal and newborn health efforts, a coherent description of project components and their intended outcomes, based on a common theory of change, can be a valuable tool. We outline an approach to developing such a tool to describe the work and the intended effect of a portfolio of nine large-scale maternal and newborn health projects in north-east Nigeria, Ethiopia and Uttar Pradesh in India. Teams from these projects developed a framework, the ‘characterisation framework’, based on a common theory of change. They used this framework to describe their innovations and their intended outcomes. Individual project characterisations were then collated in each geography, to identify what innovations were implemented where, when and at what scale, as well as the expected health benefit of the joint efforts of all projects. Our study had some limitations. It would have been enhanced by a more detailed description and analysis of context and, by framing our work in terms of discrete innovations, we may have missed some synergistic aspects of the combination of those innovations. Our approach can be valuable for building a programme according to a commonly agreed theory of change, as well as for researchers examining the effectiveness of the combined work of a range of actors. The exercise enables policymakers and funders, both within and between countries, to enhance coordination of efforts and to inform decision-making about what to fund, when and where.

**Summary box**

- Government leadership to enhance maternal and newborn health in low/middle-income countries often involves coordination of multiple diverse efforts by several projects, starting and finishing at different time points, working at different scales and in more than one geographic location.
- Characterisation is a process of describing what innovations are implemented, where, with what population and with what anticipated changes from the joint effect of all in a given geography.
- Characterisation could help policymakers, evaluators and other stakeholders understand the work of diverse actors implementing innovations with a common aim.

## Introduction

Considerable progress has been made in the field of maternal and newborn health (MNH) to generate evidence on the effectiveness of interventions that enhance maternal and neonatal survival.^1–3^ Coverage of these life-saving interventions remains limited in some settings because of low demand, bottlenecks in delivery mechanisms and poor quality of care.^4–6^ To reach targets for Sustainable Development Goal 3, a collaborative health system strengthening response is needed to address the complexity of maternal and newborn survival.^7^ The Sustainable Development Goals^8^ and global declarations and initiatives such as the Paris Declaration on Aid Effectiveness^9^ and Universal Health Coverage 2030^10^ emphasise government leadership in this response and this is well illustrated in Ethiopia where all MNH-related non-governmental organisation projects must comply with the government’s strategic plans for the health sector.

Donor support in the health field remains extensive in low/middle-income countries.^11^ Donor-funded implementation projects designed to strengthen an existing health system may work in the same geography towards the same goal but have different approaches, start and finish dates and are often not coterminous. Such support can create a complicated network of activities, which policymakers, donors, researchers and other actors must untangle if they are to engage in a well-informed way.

A rigorous, clearly structured and commonly understood description of components of implementation projects, their inputs, context, how they interact and their anticipated outcomes can help enhance coordination and inform future evaluation efforts.^12 13^ Despite numerous recommendations in the literature to apply a theory-driven approach to describing how a programme is meant to work,^14 15^ scant attention is paid to examining how inputs and anticipated outcomes are identified and mapped in practice.^16^

In this paper, we outline our theory-driven approach to characterising 61 diverse MNH innovations and mapping them onto one unifying trajectory. For us, innovations are coherent sets of activities, new to the context with a defined anticipated outcome (box 1), implemented through nine separate projects funded by the Bill & Melinda Gates Foundation. These projects operated in three geographies: north-east Nigeria, Ethiopia and Uttar Pradesh, India.^17–24^ The projects differed considerably in scope and focus and each included between 2 and 13 innovations (online supplementary appendix table A1). Our paper has two aims. First, to share the method we developed to characterise these innovations and selected results of the characterisations. We identify the contribution of each innovation to the theory of change and show their anticipated combined effect in each geography, highlighting areas of overlap and gaps in provision. Second, we share lessons learnt on the value and limitations of our approach to characterising MNH innovations.

10.1136/bmjgh-2019-001405.supp1 Supplementary data

Box 1 **Glossary**

*Characterisation*: A process of describing innovations using a framework of predefined questions.

*Direct and indirect effect* *s* *of innovations*: Innovations may have a direct effect on coverage of life-saving interventions, such as training front-line workers in community-based administration of antibiotics or family members in thermal care of the newborn. Others may have an indirect effect, promoting health-seeking behaviour such as facility delivery or postnatal checks.

*Front* *-* *line* *workers*: Nurses, midwives, doctors, and salaried and volunteer community-based health workers, such as the *Health Extension Workers* in Ethiopia, the *Community* *Volunteers* in Nigeria, or *Accredited Social Health Activist*s in India.

*Innovation*: One or more activities enhancing contact between front-line workers and service users, with the following characteristics:

- New to the context.
- A coherent set of activities which can stand alone.
- Has a defined anticipated outcome to enhance uptake of recommended maternal and newborn health (MNH) care practice by a family member (eg, thermal care of the newborn) or by a front-line worker (eg, safe and appropriate administration of antibiotics).

*Life-saving interventions*: Evidence-based practices with a biological mechanism to improve health, for example, early initiation of breast feeding or clean delivery care.

*Routine contact*: An established practice, which was the norm prior to innovations, involving contact between families and front-line workers.

*Service* *users*: Women during pregnancy, the intrapartum and postpartum periods, newborns and family members who are recipients of care or advice on MNH and in a position to act on that advice, for example, by providing clean cord care or offering advice on breast feeding.

Throughout, we illustrate the work using a case study of the Society for Family Health Maternal and Newborn Health (SFH-MNH) project in Gombe State, north-east Nigeria.

## The study

Between 2011 and 2014, we examined nine large-scale projects funded by the Bill & Melinda Gates Foundation operating in three geographies: Gombe State of north-east Nigeria; the four most populous regions of Ethiopia—Amhara, the Southern Nations, Nationalities and Peoples’ Region, Oromia and Afar; and in Uttar Pradesh, the most populous state in India. Their aim was to support their respective governments in providing community-based MNH care. Together, they targeted over 23 million people living in areas of high maternal and newborn mortality with low uptake of professional services. The combination of high population, high mortality and low uptake of services meant that there was potential for substantial impact on health.

### The country context

The maternal mortality ratio for Nigeria was estimated at 576/100 000 live births for the period 2006–2013 and is likely to be higher in the north-east since associated health indicators such as uptake of antenatal, delivery and postnatal care are poorer in this zone.^25^ The neonatal mortality rate in north-east Nigeria in this period was estimated at 43/1000 live births. There was no community-based health outreach in rural areas and ‘Community Health Extension Workers’ provided reproductive healthcare in community health centres. Only 49% of women received antenatal care from a skilled provider and 20% delivered in a facility. Thirty-two per cent of mothers and 10% of newborns had a postnatal check within the recommended 2 days of birth.^25^

In Ethiopia, the pregnancy-related mortality ratio estimate for 2009–2016 was 412/100 000 live births, and the neonatal mortality rate in the period 2012–2016 was 29/1000 live births.^26^ The greatest burden fell on the rural poor and health posts were established in each subdistrict, or *kebele,* staffed by two ‘Health Extension Workers’ who provided antenatal, delivery and postnatal care and were responsible for appropriate and timely referral where needed to Primary Health Centres, the next tier of the health service.^27^ Over the same period, in rural areas 58% of women had any antenatal care from a skilled provider, 20% delivered in a facility and 13% of mothers and 10% of newborns had a postnatal check within the recommended 2 days of delivery.^26^

In Uttar Pradesh, between 2009 and 2012, the maternal mortality ratio estimate was 258/100 000 live births and the neonatal mortality rate was 49/1000 births.^28^ Eighty-five per cent of women received some antenatal care.^28^ In the period 2011–2016, 68% of women delivered in a facility and 59% had a postnatal check within the recommended 2 days of delivery.^29^ In contrast, 29% of newborns were reported to have been checked in their first 2 days of life. Three cadres were responsible for community-based reproductive healthcare. Accredited Social Health Activists and Anganwadi Workers undertook outreach and home-based counselling and record-keeping while Auxiliary Nurse Midwives offered facility-based care.

### The portfolio of projects and innovations

All nine projects sought to support government efforts to improve MNH and stimulate adoption of health-enhancing behaviour. During this study, they were implementing 61 innovations which were either designed and developed for a specific context, such as ‘Links with pastoralist or remote communities’ of the SFH-MNH project in north-east Nigeria,^20^ or adapted to address local needs from an existing tool, such as the ‘Safe Childbirth Checklist’ of the Better Birth project in Uttar Pradesh.^23^

**Figure 1:**
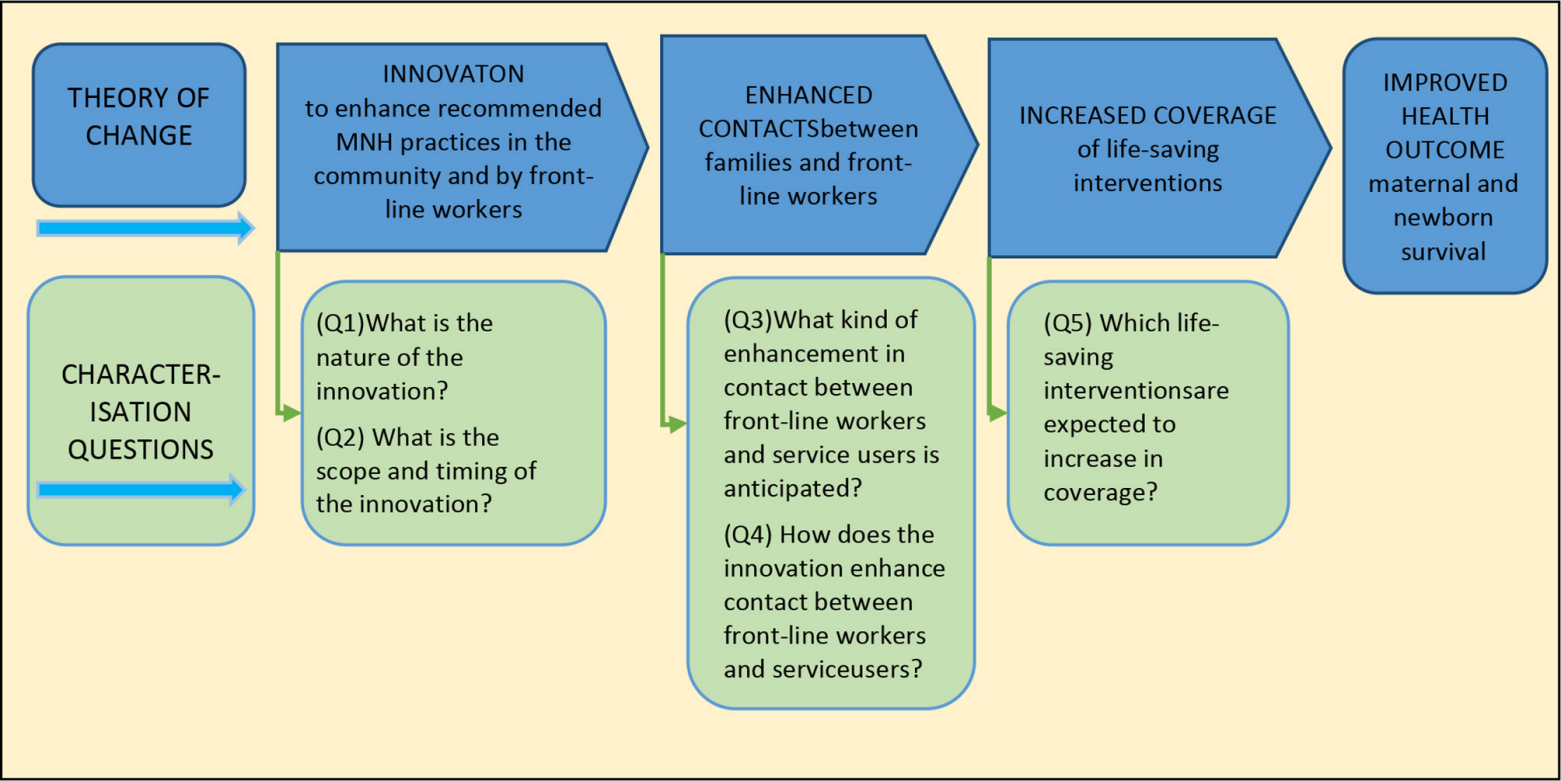
Theory of change with agreed characterisation framework questions. MNH: maternal and newborn health.

The innovations had been developed in line with an overarching theory of change from the funder, the Bill & Melinda Gates Foundation (figure 1).^30^

This theory proposed that innovations should enhance routine contacts between front-line workers and service users by making them more frequent, better quality and more equitable. These enhanced routine contacts were expected to increase coverage of evidence-based, life-saving interventions such as immediate and exclusive breast feeding or appropriate and safe administration of antibiotics, and thereby to improve the survival of mothers and newborns.^3^ The theory of change also included a scale-up loop, which described adoption of innovations beyond the project areas. Here we focus on implementation in the project areas. Evaluation of project outcomes as well as scale-up of innovations are reported elsewhere.^23 31–39^

### Glossary

Key terms used in this paper are defined in box 1.

## Developing the characterisation framework

We developed four steps to characterise the work of the MNH innovations according to the theory of change. Representatives of all projects in the portfolio met and the idea of characterising innovations was introduced, discussed and a way forward was agreed.

### Characterisation step 1: develop a characterisation framework

Characterisation teams were formed for each project, comprising a group of two to five project personnel at management level with specialist implementation or evaluation knowledge of the project, and two from the study team.

Through an iterative process, a common set of five questions was agreed that would describe innovations and how they were designed to enhance MNH. This set of questions, known as the characterisation framework, was mapped onto the theory of change (figure 1). The characterisation framework provided a mechanism to add specificity to the theory of change. The questions captured detail about each innovation: where, when and how it was expected to enhance contact with the health system, and how this enhancement was expected to lead to an improved health outcome.

### Characterisation step 2: populate the characterisation framework

Characterisation teams drew on project documents and critical discussion to agree a set of innovations that represented their work. Critical discussion was also essential to fill gaps in project documentation and agree a definition of each innovation. Projects with external evaluation partners, such as Better Birth and Manthan in Uttar Pradesh, had well-defined innovations, while other projects such as the Uttar Pradesh Community Mobilisation Project (UP-CMP) had not previously considered distinct innovations within its overall project.

The characterisation teams from the nine projects identified 61 innovations (online supplementary appendix table A1) across the study period. These had varied intended outcomes such as enhanced MNH care seeking, access and provision of MNH services and adoption of healthy MNH-related practices in the home and community.

Each innovation was described using standardised terms to address the five agreed questions in the characterisation framework (figure 1). An example of a completed characterisation of the SFH-MNH project in north-east Nigeria is given in online supplementary appendix tables A2 and A3. The five questions were as follows:

#### (Q1) What is the nature of the innovation?

This was captured by an outline narrative, a statement of purpose and a description of the main implementation activities, described in three categories: develop (eg, materials, training package); equip (eg, equipment, toolkit); train and support (eg, supervision, refresher training).

#### (Q2) What is the scope and timing of the innovation?

This described the target population and geographical focus, as well as the start and end dates of the innovations.

#### (Q3) What kind of enhancement between front-line workers and service users is anticipated as a result of the innovation?

The anticipated enhancement in contacts between front-line workers and service users was documented in terms of frequency, quality and equity.

#### (Q4) How did the innovation enhance contacts between front-line workers and service users?

Characterisation teams documented routine contacts between each type of front-line worker and service user and specified the combination of innovations that were to enhance those contacts. To illustrate, online supplementary appendix table A3 shows that in 2014 SFH-MNH sought to enhance the work of four different types of front-line workers in north-east Nigeria: traditional birth attendants, community volunteers, call centre staff and proprietary patent medicine vendors. Routine contacts between the tradition birth attendants and pregnant women involved identifying a pregnant woman in the community and advising on antenatal care. Four of SFH-MNH’s nine innovations enhanced this contact: innovation 1—mapping of service users and provision; innovation 2—train and deploy traditional birth attendant; innovation 4—front-line worker toolkit; innovation 8—mass media event. The expected changes in practice following these innovations were: more complete and timely identification of pregnant women; registration for antenatal care; enhanced delivery of key MNH messages; enhanced detection of danger signs; and appropriate referral.

#### (Q5) Which life-saving interventions are anticipated to increase in coverage?

Building on the understanding of anticipated changes in contacts, each characterisation team identified the resulting expected changes in coverage of life-saving interventions and whether these were affected directly or indirectly. Drawing again on the illustrative example of SFH-MNH (online supplementary appendix table A3), the enhanced contacts between traditional birth attendants and pregnant women had an effect on coverage of life-saving interventions delivered during antenatal care. This was indirect because the traditional birth attendant could advise or refer women to antenatal care but did not directly deliver life-saving antenatal interventions herself. In contrast, enhanced contact between the traditional birth attendant and a woman in labour at home could have a direct effect on coverage of life-saving interventions through a clean delivery kit and hand-washing with soap.

### Characterisation step 3: collate information from all characterisation teams by geography

All innovations operating throughout the study are listed in online supplementary appendix table A1. We collated characterisations by geography to describe the anticipated combined population-level effects of all innovations in that geography on contacts during antenatal, delivery and postnatal care and on coverage of life-saving interventions.

The theory of change proposes that innovations enhance routine contacts between front-line workers and service users in each geography. To illustrate, we examined anticipated enhancement of skilled birth attendance, focusing on five dimensions: frequency; quality as timeliness; quality as content of care; equity of access; and facility readiness (table 1). Not all projects implemented innovations to address all five dimensions. In Ethiopia, all three projects included innovations which were contributing to enhanced frequency and timeliness of skilled birth attendance through their community-based innovations to enhance awareness of MNH and care seeking, as well as strengthening the work of community front-line workers. None was actively working on facility readiness. In Uttar Pradesh, only the Better Birth project sought to enhance facility readiness through its Safe Childbirth Checklist innovation. Better Birth and Manthan both sought to enhance quality of skilled birth attendance in Uttar Pradesh. The same innovation mapping was undertaken to describe antenatal and postnatal care.

**Table 1:** Table showing whether projects anticipate enhancement in frequency, quality and equity of skilled birth attendance at community and primary level during the study period (Y, yes; N, no)

Project and locations	Type of enhancement in routine contact				
	Frequency of skilled birth attendance	Quality of skilled birth attendance (timeliness)	Quality of skilled birth attendance (content)	Equity of access to skilled intrapartum care	Facility readiness (equipment infrastructure)
Gombe State, north-east Nigeria					
SFH-MNH	Y	Y	Y	Y	Y
SAQIP	N	N	N	N	N
Four regions in Ethiopia: Amhara; Southern Nations, Nationalities and Peoples’ Region; Oromia and Afar					
L10K	Y	Y	Y	Y	N
COMBINE	Y	Y	Y	N	N
MaNHEP	Y	Y	N	Y	N
Uttar Pradesh					
Manthan	Y	Y	Y	Y	N
UP-CMP	Y	Y	N	Y	N
Better Birth	N	Y	Y	N	Y
Sure Start	Y	Y	N	Y	N

SFH-MNH, Society for Family Health Maternal and Newborn Health; SAQIP, State Accountability for Quality Improvement Project of PACT Nigeria; L10K, Last 10 Kilometers Project of JSI Research and Training Institute; COMBINE, Community-Based Interventions for Newborns in Ethiopia–Saving Newborn Lives; MaNHEP, Maternal and Newborn Health Extension Program, Emory University; Manthan, Manthan Project–IntraHealth International; UP-CMP, Uttar Pradesh Community Mobilisation Project, Public Health Foundation of India; Better Birth, Better Birth Project, Ariadne Labs, Brigham and Women’s Hospital and Harvard TH Chan School of Public Health; Sure Start, Sure Start Project of PATH.

Following the theory of change (figure 1), enhanced contacts between front-line workers and service users would result in increased coverage of life-saving interventions. The effect may be direct or indirect (see box 1 for definitions). To illustrate, we focus on the intrapartum period, described in table 2.

**Table 2:** Anticipated effect on coverage of life-saving interventions at birth, deliverable at community and primary level, by project

Projects and locations	Life-saving interventions				
	Appropriate administration of antibiotics	Management of postpartum haemorrhage using uterine massage and uterotonics	Active management of the third stage of labour	Hand-washing with soap, use of gloves by delivery attendant	Management of early onset of labour using corticosteroids
Gombe State, north-east Nigeria					
SFH-MNH	I	I	I	D	I
SAQIP	I	I	I	I	I
Four regions in Ethiopia: Amhara; Southern Nations, Nationalities and Peoples’ Region; Oromia; Afar					
L10K	I	I	I	D	I
COMBINE	I	I	I	I	I
MaNHEP	I	I	I	D	I
Uttar Pradesh					
Manthan	D	D	D	D	I
UP-CMP	I	I	I	I	I
Better Birth	D	D	D	D	D
Sure Start	I	I	I	I	I

SFH-MNH, Society for Family Health Maternal and Newborn Health project; SAQIP, State Accountability for Quality Improvement Project of Pact, Nigeria; L10K, Last 10 Kilometers Poject of JSI Research and Training Institute; COMBINE, Community-Based Interventions for Newborns in Ethiopia – Saving Newborn Lives; MaNHEP, Maternal and Newborn Health Extension Program, Emory University; Manthan, Manthan Project – IntraHealth International; Better Birth, Better Birth Project, Ariadne Labs, Brigham and Women’s Hospital and Harvard T.H.Chan School of Public Health; UP-CMP, Uttar Pradesh Community Mobilisation Project, Public Health Foundation of India; Sure Start, Sure Start Project - PATH.

In north-east Nigeria and Ethiopia, the anticipated joint effect of the innovations was largely indirect, mainly comprising promotion of facility delivery, recognition of danger signs and appropriate timely referral, while in Uttar Pradesh, Better Birth and Manthan worked on enhancing coverage through engagement with front-line workers who were directly administering life-saving interventions and the facilities in which they worked.

An important finding of this step of the characterisation process was that the implementation of the innovations differed by time and geography, even within a single project. To examine the potential joint effect of the projects in any one geography, information was collected on the timing and geographical focus of each innovation. A high-level summary of timing of projects is shown in table 3. As the table shows, while the projects in north-east Nigeria and Ethiopia did overlap in terms of timing, in Uttar Pradesh, only UP-CMP and Better Birth operated in the same period. Examination of data on geographical focus suggested a mixed picture. In north-east Nigeria, both projects intended to work in the whole of Gombe State so there was an opportunity for synergy in the field, while in Ethiopia the three projects operated in different woreda (districts) so there was no such opportunity.

**Table 3:** Planned timelines for projects in Gombe State, Nigeria, four regions in Ethiopia and Uttar Pradesh, India.

	2009		2010		2011		2012		2013		2014		2015
Gombe State, Nigeria													
SFH-MNH													>
SAQIP													>
Four regions in Ethiopia: Amhara; Southern Nations, Nationalities and Peoples’ Region; Oromia; Afar													
L10K	<												>
COMBINE													
MaNHEP													
Uttar Pradesh													
Manthan													
UP-CMP													
Better Birth													>
Sure Start	<												

Key: < started before 2009; > continued beyond 2015.

Grey area: implementation period

SFH-MNH, Society for Family Health Maternal and Newborn Health project; SAQIP, State Accountability for Quality Improvement Project of Pact, Nigeria; L10K, Last 10 Kilometers Poject of JSI Research and Training Institute; COMBINE, Community-Based Interventions for Newborns in Ethiopia –Saving Newborn Lives; MaNHEP, Maternal and Newborn Health Extension Program, Emory University; Manthan, Manthan Project – IntraHealthInternational; UP-CMP, Uttar Pradesh Community Mobilisation Project, Public Health Foundation of India; Better Birth, Better Birth Project, Ariadne Labs, Brigham and Women’s Hospital and Harvard T.H. Chan School of Public Health; Sure Start, Sure Start Project of PATH.

### Characterisation step 4: annual update

The characterisations were updated annually to keep abreast of changes in project design and implementation and to inform evaluation. Substantial changes took place, for example, when SFH-MNH Nigeria embarked on their work in 2012 they anticipated implementation of 13 innovations in two states (online supplementary appendix table A1), but by the 2014 update they were implementing nine innovations in one state (online supplementary appendix table A2).

## Lessons learnt

Based on meeting notes, internal discussions and reflection, we identified a number of lessons of value to policymakers, donors, researchers and evaluators, and implementers when considering using our approach.

### Value of using a theory of change

Mapping innovations onto an established overarching theory of change enabled stakeholders including donors, policymakers and managers and evaluators of projects to identify what innovations were implemented, where and when. At each step, we identified the contribution of every innovation within each project and the anticipated change resulting from the combined work of all projects. This enabled us to apply common measures, including frequency, quality and equity of targeted contacts, and coverage of targeted life-saving interventions (figure 1). We were also able to identify gaps in provision and areas of overlap, which were of interest to policymakers, the funder and the implementers in each geography.

A theory of change can be defined in many ways.^14 40^ It can be seen as an iterative and participatory tool to describe a programme fully, examine assumptions and foster learning through reflection and revision,^41^ or it can also be used more narrowly to describe a logical programme pathway from inputs to outcome and provide a framework for evaluation.^42^ We used an existing theory of change in this latter way to make sense of a portfolio of diverse innovations implemented within existing health systems in three geographies, and identified their anticipated individual and joint contribution to enhancing MNH.

### Value for characterisation teams

Our study made real a rather abstract overarching theory of change. For some participating project staff, the exercise gave them an opportunity to engage in assessing how their work contributed to this overarching theory, thereby giving them a sense of common purpose within the donor’s broader strategy to improve MNH. Project teams were interested to learn from each other’s work at annual meetings, which enabled them to identify both commonalities and uniqueness, as well as considering the adoption of common indicators, which could be used to measure the combined effects of multiple innovations across each geography. The discussions stimulated the teams to examine assumptions about steps along the pathway from innovation to outcome and generated a new interest in process evaluation and in qualitative work to investigate the complexities of their work.

### Defining innovations

Defining innovations was a challenge for some projects, which proposed many components as individual innovations, such as SFH-MNH’s front-line workers toolkit, while others proposed fewer components, such as Better Birth’s introduction of a Safe Childbirth Checklist in health facilities. Focusing on the feasibility of measuring the outcome of the innovation encouraged these projects to consider the purpose of each innovation and how it might interact with others to affect an outcome.

### Time needed to complete the characterisations

Of the nine projects, two did not have a well-developed evaluation plan or external evaluation partners: they found it challenging to conceptualise their work in terms of discrete innovations with measurable outcomes. For these projects, characterisation meetings took up to 2 days. The other seven projects did have external evaluation partners, well-articulated innovations and measurable outcomes, and for these the exercise could be completed within 2 hours. The most efficient characterisation teams in terms of completeness, clarity and speed included three to five members of project staff who had an operational understanding of the details of the innovations and an overview of the purpose of their work.

### Engaging with complexity

Our work characterised innovations by considering them as sets of bounded activities with interacting and mutually reinforcing components, developed for or adapted to the context. As such, they could be seen as complex interventions.^43^ However, in reality projects in the same geography operated at different times or different places, to different scales, thereby losing opportunities for synergy. For example, Manthan and Better Birth both worked on enhancing the quality of skilled birth attendance in Uttar Pradesh. However, Manthan worked with Auxiliary Nurse Midwives in one block in Bahraich District and Better Birth implemented the checklist in 60 hospitals in 24 districts in the State.

Systems thinking examines interactions between the innovation and the whole system, rather than between the innovation and one component of the system. It examines how the system adapts and measures outcomes at all levels, and considers how outcomes in different parts of the system affect one another and the mechanisms by which the effect of an innovation is amplified or diminished.^44^ This approach would be suited to a set of projects aligned and integrated in both space and time, and we were not able to adopt it here.

We engaged with the complexity of individual projects by examining how the innovations operated, and their effect on front-line workers and targeted improvements in the health system in different combinations and different contexts. The projects were dynamic and were reshaped in response to changing needs. We updated our characterisations annually to capture these changes. Further examination of complexity would involve a separate qualitative study.

### Harmonised terms

Behaviour change interventions in the literature tend not to have commonly agreed terms and definitions,^12 45^ in contrast to natural science experiments.^46 47^ Terms and definitions can lack precision and standardisation so knowledge in the field of behaviour change in healthcare seeking and provision has been slow to evolve, hampering scale-up of effective initiatives.^12^ Particularly where there are numerous stakeholder groups, such as government policymakers, implementers, donors and researchers and evaluators, a common vocabulary has been essential. Those wishing to coordinate multiple efforts under a common set of criteria, harmonise and align support to government priorities^9^ or replicate initiatives need a lucid description of what has been tried and a way to compare different initiatives, including an assessment of their contribution to behaviour change.^12 16 48–50^ This clarity is particularly valuable where ministries of health and other health authorities deal with multiple donors and implementing partners. We used harmonised terms across projects to describe individual and combined efforts to improve MNH, whether and how they interact, their location, timing and what changes were made across all projects in a given geography. We would advocate for such an approach in any group seeking to compare implementation or effectiveness of a set of complex interventions.

### Limitations

Our study had a number of limitations. First, we did not examine the extent to which the innovations were relevant for the purpose they set out to address. In part, this is because the projects were already under way when we undertook the characterisations.

Second, we included generalised material on the health system in each country to provide the context for the characterisations. On reflection, we could have described the context in a more granular way,^13^ including other substantive activities taking place in the same geographies, to enable us to consider how the innovations and the system adapt in different contexts and help explain findings in different contexts.

Third, there was risk of reporting bias in the characterisations, as characterisation teams may have downplayed programmatic challenges.^51^ Although we made efforts to ensure that the opinion of one team member did not dominate at the expense of others, we were not in a position to verify this objectively or to assess whether descriptions were accurate.

Fourth, as part of the characterisation process, more attention could have been paid to the length of time needed for an innovation to have a measurable effect on outcomes. Innovations such as SFH-MNH’s work to enhance supplies of clean delivery kit were likely to have an immediate and direct effect on health outcomes because of improved hand-washing and use of gloves during delivery. Others were only likely to have an effect on outcomes in the longer term, such as changing community attitudes through the mass media event.

Lastly, for projects without predefined innovations, discussions were lengthy and resulted in relatively large numbers of separate innovations. By focusing on capturing and describing discrete innovations, our approach may have missed synergistic aspects of combinations of these innovations.

## Conclusions

Development assistance has grown considerably in the last 20 years and there is concern that ministries of health in low-income countries manage support for their national health strategies from multiple and diverse funders, who may not always respond to country needs.^11 51^ Creating a standardised characterisation of the work of donor-funded implementers supporting government health authorities could be a valuable operational tool to describe who is doing what, where and when, and can highlight whether and how externally funded projects are harmonised with each other and aligned with country priorities and programmes. Such a tool can also be valuable to donors and implementers who are interested in harmonising efforts of multiple actors and building a programme according to a commonly agreed theory of change, as well as to researchers and evaluators examining the effectiveness of the combined efforts of a set of projects and innovations. The exercise enables policymakers and funders, both within and between countries, to enhance coordination of efforts and to inform decision-making about what to fund, when and where.
