# Supplementary material for: Characterising innovations in maternal and newborn health based on a common theory of change: lessons from developing and applying a characterisation framework in Nigeria, Ethiopia and India
Source: BMJ Glob Health. 2019 Jul 18;4(4):e001405. doi: 10.1136/bmjgh-2019-001405 (PMC6666810; doi:10.1136/bmjgh-2019-001405)
Supplement: Supplementary data [file bmjgh-2019-001405supp001.docx]

**Appendices: Table of innovations and Characterisation of innovations: Society for Family Health Maternal and Newborn Health project, Gombe State**

**Table A1. Project innovations**

| Table A1. Project innovations 2011-2014, by geography. | | | | | | | | |
| --- | --- | --- | --- | --- | --- | --- | --- | --- |
| **Gombe State Nigeria project innovations** | | **Ethiopia project innovations** | | | **Uttar Pradesh India project innovations** | | | |
| Society for Family Health | PACT | Last 10 Kilometres | MaNHEP | SNL COMBINE | Better Birth | Manthan | Sure Start | UP-Community Mobilisation Project |
| 1. Mapping of service users and provision; 2. Train and deploy skilled Traditional Birth Attendants 3. Train and deploy FOMWAN community volunteers 4. Frontline workers’ toolkit; 5. Financial incentives for frontline workers; 6. Call centre; 7. Emergency Transport Scheme; 8. Mass media event; 9. Enhanced clean delivery kit supplies 10. Advocacy training for men; 11. Forum of mothers-in-law; 12. Upgrade primary care facilities; 13. Links with pastoralist / remote communities. | 1. Institutional Strengthening; 2. Capacity development for Frontline Organisations; 3. Enhance MNCH value network; 4. Enhance Civil Society Organisation/ Government Collaboration 5. Build and support a TBA Forum. | 1. Family conversations; 2. Participatory community quality improvement; 3. Community solutions fund; 4. Anchors; 5. Health Extension Worker training and supervision; 6. Health Development Army training; 7. Non-financial incentives for Health Development Army volunteers; 8. Family Health Card; 9. Improved referral linkages; 10. Community-based data for decision-making. | 1. Behaviour change communication strategy; 2. CMNH Family Meetings, community gatherings. 3. MNH care package; 4. Frontline worker team development; 5. Performance monitoring for quality improvement. | 1. Enhance MNH profile in the community; 2. MNH awareness in community structures 3. Health Development Army training; 4. Health Extension Worker training; 5. Community- based administration of antibiotics. 6. Health Extension Worker / Health Development Army linkages. | 1. Safe Childbirth Checklist 2. Safe Childbirth Checklist training and supervision. | 1. Auxiliary nurse-midwife training in skilled birth attendance 2. mSakhi phone app; 3. mSahki home based newborn care phone app 4. Emergency transport scheme 5. Mother and child tracking system. | 1. 1^st^ hour campaign; 2. Mothers’ groups; 3. Letter to my father 4. Revitalise Village Health & Sanitation Committees 5. Accredited Social Health Activist (ASHA) mentoring and supportive supervision 6. Recognise good performance of ASHAs and Village Health and Sanitation Committees 7. ASHA toolkit ­ 8. NGO partnership architecture. | 1. Diffusion of MNCH practice; 2. Capacity building of the self-help groups / federations in RMNCHN. 3. Capacity building of Self-Help Groups (SHGs) and federations; 4. SHG-health system links; 5. Scale-up model; 6. Community-managed monitoring, learning and evidence-based decision-making; 7. Self Help Group - health system links. |

| **Table A2. Characterisation Questions 1, 2 and 3. Society for Family Health, 2014 update.** | | | | |
| --- | --- | --- | --- | --- |
| **Innovation** | Q1. What is the nature of the innovation? | | | Q2. What is the scope and timing of the innovation? |
|  | **Description** | **Purpose** | **Project Activities** |  |
| 1. Mapping of service users and provision | Innovation to enhance existing government mapping of facilities to include element of quality of care (approved facilities).  Maps & database of approved facilities & trained FLWs made available to Call Centre staff.  FLWs given information on closest appropriate approved facilities.  Map telecom coverage in intervention area. | To enhance quality of referral, by making mapping information available to FLWs, families and the community on appropriate referral facilities in the area.  To increase contact with FLW by informing families / community of trained FLW in the area.  To enhance effectiveness and efficiency of referral.  To ease access to the Call Centre each community has appropriate SIM card for free-phone contact. | **Develop:** Maps and a database for use by Call Centre staff, TBAs and FOMWAN.  **Equip:** FLWs with community-specific information; Call Centre staff with maps and a database of approved facilities and trained FLWs.  **Train and support:** Call Centre staff in using the maps and database; FLWs in using community-specific information; On-going support by up-dating database every 2 years  FLW up-dated on new information through supervision and quarterly review meetings. | Whole of Gombe State. Started 2010, ongoing. |
|  |  |  |  | Q3. What type of enhancement in contacts is anticipated as a result of the innovation? |
|  |  |  |  | **Frequency:** Access to Call Centre and thereby to referral facilities, access to nearby, trained FLWs through the Call Centre  **Equity:** New information is available on location of pastoralist / remote communities, which might otherwise not access care. |
| 2. Train and deploy skilled Traditional Birth Attendants (rural) | Prepare and deploy a community-based cadre of rural FLW to  * promote ANC, identify danger signs in pregnancy and refer appropriately  * offer clean delivery care, identify danger signs and facilitate appropriate referral  * undertake post-partum checks of the mother and newborn, identify danger signs and facilitate appropriate referral. | To increase contact between FLW and pregnant, delivering and postnatal women and newborns in all communities.  To enhance the cleanliness of home-based delivery care.  To enhance identification of danger signs in pregnancy, delivery and post-partum and refer in a timely and appropriate way.  To increase and enhance coverage of post-natal checks and appropriate referral of the mother and baby. | **Develop:** Training and deployment programme; training manual; recording system; supervision and support system.  **Equip:** Project staff have training manual and recording system; FLWs have the toolkit including appropriate data-collection tools (see SFH innovation 4); uniform for easy identification.  **Train and support:** FLWs have 5 days' training (in Phase 2 increased to 6 days) followed by monthly supervision visits by project staff; quarterly review meetings with MoH and SFH management. | Q2. What is the scope and timing of the innovation? |
|  |  |  |  | Phase I: initially with six Local Government Areas – June 2010 ongoing. Phase II: All rural Gombe State – September 2010 ongoing. |
|  |  |  |  | Q3. What type of enhancement in contacts is anticipated as a result of the innovation? |
|  |  |  |  | **Frequency:** FLWs generate interest in MNH, promote facility care  **Quality:** Enhanced MNH knowledge and practice including delivery care (TBAs)  **Equity:** FLWs deployed in under-served communities |

| **Table A2. continued. Characterisation Questions 1, 2 and 3. Society for Family Health, 2014 update.** | | | | |
| --- | --- | --- | --- | --- |
| **Innovation** | Q1. What is the nature of the innovation? | | | Q2. What is the scope and timing of the innovation? |
|  | **Description** | **Purpose** | **Project Activities** |  |
| 3. Train and deploy community volunteers (urban) | Prepare, train and deploy community-based urban FLW to  * promote antenatal care and facility deliveries, key messages on clean birth practices, effective post-partum care of the mother and newborn.  * identify danger signs and facilitate appropriate referral (using Call Centre and Emergency Transport Scheme).  * undertake post-partum checks of the mother and newborn. | To increase and enhance home-based contact between FLW and families in all communities.  To raise awareness of key MNH messages.  To enhance appropriate referral for pregnant and delivering women and for newborns.  To increase demand for facility-based antenatal and PN care  To increase the proportion of facility deliveries  To increase and enhance coverage of PP checks and appropriate referral of the mother and baby. | **Develop:** Training and deployment programme for FLWs, training manual; appropriate recording system; supervision and support system.  **Equip:** Project staff have training manual and recording system; FLWs with the toolkit including appropriate data-collection tools (see SFH innovation 4); uniform clothing items for easy identification.  **Train and support:** 3day training (to be increased to 5 days); monthly supervision visits by project staff and quarterly review meetings with MoH and SFH management. | All urban centres. June 2010 ongoing |
|  |  |  |  | Q3. What type of enhancement in contacts is anticipated as a result of the innovation? |
|  |  |  |  | **Frequency:** FLWs generate interest in MNH, promote facility care  **Quality:** FLW training enhances MNH knowledge and practice.  **Equity:** FLWs deployed in all communities including pastoralist / remote and poorly-served |
| 4. Frontline workers’ toolkit | Toolkit comprises equipment to facilitate FLW access to homes and enhance their performance:  equipment to help access in adverse weather conditions and in the dark; a flipchart of essential information for FLWs to use for FLWs' own information and to impart key messages to community members; a foot-length ruler; containers with stickers indicating key MNH events and outcomes (e.g. delivery, delivery outcome, referral, postnatal visit) in which non-literate FLWs place pebbles as a way of keeping a tally of their work, data are collected at monthly supervision meetings. | To enable FLWs to impart information to families in a standardised and well-informed way.  To help FLWs give coherent and consistent messages.  To help FLWs plan and manage home visits for MNH care.  Foot length ruler to help identify premature babies in need of special care.  Containers with stickers to enhance data collection among non-literate FLWs on number of pregnant women reached and referred, deliveries conducted, and to enhance data quality and thereby effective project management. | **Develop:** Toolkit - flipchart with MNH information for FLW, mother and family; guidance on imparting key messages effectively; cell phone, torch, umbrella, rain boots; foot-length rule for newborns; containers and pebbles to track work of less literate FLWs  **Equip**: FLWs receive toolkit; less literate FLWs receive jars with stickers to log major MNH events and outcomes  **Train and support**: FLWs in toolkit use; FLWs have monthly supervision visits by project staff and quarterly review meetings with MoH and SFH management; train less literate FLWs to collect quality data; support FLWs through feedback at monthly meetings; refresher training at quarterly review meetings. | Q2. What is the scope and timing of the innovation? |
|  |  |  |  | Each FLW in Gombe State. Started June 2010, ongoing |
|  |  |  |  | Q3. What type of enhancement in contacts is anticipated as a result of the innovation? |
|  |  |  |  | **Quality**: enhanced knowledge base of FLWs, better communication materials. |

| **Table A2. continued. Characterisation Questions 1, 2 and 3. Society for Family Health, 2014 update.** | | | | |
| --- | --- | --- | --- | --- |
| **Innovation** | Q1. What is the nature of the innovation? | | | Q2. What is the scope and timing of the innovation? |
|  | **Description** | **Purpose** | **Project Activities** |  |
| 5. Financial Incentives for FLW | Performance-based financing. A system of financial incentives / rewards for FLWs who accompany women to a facility for delivery care or who refer a woman or newborn to a facility in response to danger signs.  A parallel system of rewards to Local Government Area (LGA) recognising achievement in facility deliveries and facility-based management of referrals. | To increase proportion of women delivering in a facility.  To enhance timely and effective referral. | **Develop:** Comparative scoring system to (a) identify LGAs with good record of facility delivery; (b) FLWs with good record of accompanying women for facility delivery; facility-based system to record deliveries accompanied by FLWs; system of remunerating TBAs.  **Equip:** Facilities have stamps verifying that TBA has accompanied a woman for facility delivery. TBAs have forms to record referrals.  **Train and support:** Notify LGAs, facilities and FLWs of the scheme; train all actors in how best to record women in labour / with danger signs accompanied by FLW. | 10 of the 11 Local Government Areas in Gombe State (excludes Gombe Town). Started June 2010, ongoing. |
|  |  |  |  | Q3. What type of enhancement in contacts is anticipated as a result of the innovation? |
|  |  |  |  | **Frequency:** FLWs are rewarded for accompanying women to facilities for delivery |
| 6. Call Centre | A free-phone Call Centre staffed by trained health providers offers information to community-based providers, pregnant women and families. Links pregnant or delivering women with community-based providers and with the Emergency Transport Scheme. Promotes facility-based care.  PROSPECTIVELY to include malaria / family planning and other broader MNCH information | To make information on MNH accessible to members of all communities.  To provide information on safe practice in pregnancy, delivery and the post-partum period to women, families and FLWs.  To increase proportion of women delivering in a facility  To increase proportion of women accessing ANC and PNC  To enhance timely referral | **Develop:** Call Centre; database on trained providers; SOPs for giving information and advice; information leaflets; publicity bill-boards and cards; mass media and radio messages  **Equip:** Call Centre has reliable telephone technology; Call Centre staff has hard copies of SOPs; community has publicity and information on the Call Centre; FLWs and targeted communities have appropriate SIM cards for reliable access to the Call Centre (builds on Innovation 1: mapping).  **Train and support:** Call Centre staff in using telephone technology, database and in following SOPs; FLWs in accessing the Call Centre; Monthly and quarterly team meetings to support Call Centre staff, e.g. feedback from exit interviews and mystery callers. | Q2. What is the scope and timing of the innovation? |
|  |  |  |  | One Call Centre for Gombe, started Sept 2010, ongoing. Enhanced Call Centre prospective. |
|  |  |  |  | Q3. What type of enhancement in contacts is anticipated as a result of the innovation? |
|  |  |  |  | **Frequency:** Easier and more frequent contact with CC and other FLWs  **Quality:** By training Call Centre staff, information and advice for families and FLWs is improved, enhanced response to danger signs - appropriate and timely referral |

| **Table A2. continued. Characterisation Questions 1, 2 and 3. Society for Family Health, 2014 update.** | | | | |
| --- | --- | --- | --- | --- |
| **Innovation** | Q1. What is the nature of the innovation? | | | Q2. What is the scope and timing of the innovation? |
|  | **Description** | **Purpose** | **Project Activities** |  |
| 7. Emergency Transport Scheme (ETS) | Scheme run through the NURTW^[[1]](#footnote-1)^ officers to offer low-cost or free transport to a referral centre. Mobile Park Managers train volunteer drivers in: lift and transport women, use standard reporting format; quick repairs to vehicles; traffic regulations.  Trained drivers become peer educators, train and recruit additional drivers. Families, FLWs^2^ or the Call Centre can contact the drivers. Some have car stickers for easy ID. Drivers rewarded by priority loading at transport park, sometimes fuel, community appreciation. | To facilitate timely access to the appropriate referral centre for normal delivery or in response to danger signs (mother and newborn).  To render facility-based care accessible to members of all communities | **Develop:**  ETS system in collaboration with NURTW^1^; Management Information System; ETS training curriculum and training materials; job-aid for drivers; log-book; IEC materials; car stickers  **Equip:** Drivers with IEC materials, stickers and information packs; mobile park managers with recording documents; peer educators with training materials/ log books  **Train and support:** Inform NURTW, mobile park managers and drivers about the scheme; train drivers in safe MNH; train and support peer-educators to pass on MNH information and to recruit new drivers | At least one transport park per Local Government Area in Gombe State. Started Sept 2010, ongoing. |
|  |  |  |  | Q3. What type of enhancement in contacts is anticipated as a result of the innovation? |
|  |  |  |  | **Frequency:** Women / newborns with complications reach facility  **Equity:** Free service, can benefit most remote communities |
| 8. Mass media event | SFH works through an agency to develop and air radio spots to promote MNH concepts, the Call Centre and facility delivery.  Billboards promote the Call Centre and publicise contact telephone numbers among community member.  Mid-mass media, leaflets & posters on Call Centre, publicise in the community, key messages on MNH | To promote the work of the Call Centre and the Emergency Transport Scheme.  To promote key MNH messages and facility delivery.  To enhance the community’s knowledge base on MNH. | **Develop:** Mass media event including radio messages / jingles, bill-boards, posters, stickers and leaflets.  **Equip:** Radio houses with recorded jingles; FLWs and Emergency Transport Scheme drivers with stickers and posters; at bi-annual the MNCH week distribute posters, leaflets and stickers.  **Train and support:** FLWs are trained in how to use new communications materials. | Q2. What is the scope and timing of the innovation? |
|  |  |  |  | All Gombe State. September 2010, ongoing. |
|  |  |  |  | Q3. What type of enhancement in contacts is anticipated as a result of the innovation? |
|  |  |  |  | **Frequency:** Increased awareness and more use of Call Centre, and thereby FLWs and ETS  **Equity:** Use of radio - can be heard by everyone, no cost, most benefit to the poor |
| 9. Access to cheaper clean delivery kits | A social marketing innovation to make Clean Delivery Kits available at reduced cost through TBAs and FOMWAN volunteers (and Patent medicine vendors in the learning phase). | To enhance clean delivery when delivery takes place in the home, through the use of clean delivery kits. | **Develop:** Revised clean delivery kit package; training guide and reference material for each FLW cadre (Patent medicine vendors, TBAs, and FOMWAN).  **Equip:**  FLWs with the clean delivery kit and guides.  **Train and support:** As part of routine FLW training, train in the use of the clean delivery kit and reference material; Support TBAs, and FOMWAN volunteers by following up during review meetings; **m**onitor distribution of clean delivery kits and give feedback. | Q2. What is the scope and timing of the innovation? |
|  |  |  |  | Every Local Government Area. Phase I June 2010 – 2012. Phase II: 2012 – 2014 |
|  |  |  |  | Q3. What type of enhancement in contacts is anticipated as a result of the innovation? |
|  |  |  |  | **Frequency:** People will buy the clean delivery kits if they are less expensive - more contact with Patent medicine vendors  **Equity**: Poorest least likely to afford/ access standard-cost clean delivery kits |

| **Table A3.** **Characterisation Society for Family Health, Nigeria, 2014 update.** Contacts between frontline workers and service users, showing: (Q4) How does the innovation enhance contact between frontline workers and service-users? and (Q5) Which life-saving interventions are anticipated to increase in coverage? | | | | | |
| --- | --- | --- | --- | --- | --- |
| **Contact between the Traditional Birth Attendant (TBA) and service-users** | | | | | |
|  | **Traditional birth attendant and pregnant women** | **Traditional birth attendant and women in labour** | **Traditional birth attendant and new Mothers** | **Traditional birth attendant and newborns** | **Traditional birth attendant and husbands/Families** |
| **Routine contact ->** | ID pregnancy, informal ANC | Delivery assistance | Occasional postpartum visit | Occasional postnatal visit (rarely) | Occasional contact |
| **Innovations enhancing this contact->** | 1. Mapping of service users and provision; 2. Train and deploy TBA; 4. FLW toolkit; 8. Mass media event. | 1. Mapping of service users and provision; 2. Train and deploy TBA; 4.FLW toolkit; 5. Financial incentives; 6. Call Centre; 7. Emergency Transport Scheme; 9. Enhanced clean delivery kit supplies. | 1. Mapping of service users and provision; 2. Train and deploy TBA; 4.FLW toolkit; 6. Call Centre; 7. Emergency Transport Scheme. | 2. Train and deploy TBA; 4. FLW toolkit; 5. Financial Incentives; 6. Call Centre; 7. Emergency Transport Scheme; 9. Enhanced clean delivery kit supplies. | 1. Mapping of service users and provision; 2. Train and deploy TBA; 4.FLW toolkit; 6. Call Centre; 7. Emergency Transport Scheme; 8. Mass media event. |
| **Expected change in practice ->** | More complete and more timely identification of pregnant women and registration for ANC. Enhanced delivery of key messages. Enhanced detection of danger signs and appropriate referral. | More complete and timely referral to appropriate delivery facility. If TBA assists home delivery, appropriate use of clean delivery kit. Enhanced detection of danger signs and appropriate referral. | More complete and timely postpartum visit. Enhanced detection of danger signs, timely referral to appropriate facility if needed. | More complete and timely postnatal visit. Enhanced detection of danger signs, timely referral to appropriate facility if needed. | More engagement of husbands and families in care at all stages |
| **Expected effect on coverage of life-saving interventions (Direct or Indirect) ->** | INDIRECT: Identification of danger signs and appropriate referral. As a result, increased coverage of TT vaccine; Iron supplementation; Prevention of malaria; prevention and management of STIs including syphilis and HIV | DIRECT: Use clean delivery kit appropriately, e.g. hand washing with soap and use of gloves by delivery attendant.  INDIRECT: identify danger signs and appropriate referral, promote facility delivery, so prophylactic uterotonics to prevent PPH; management of PPH; AMTSL; access to c-section | DIRECT: Home-based PN care: detect and treat anaemia  INDIRECT: ID danger signs and appropriate referral, e.g. postpartum sepsis. | DIRECT: Home-based postnatal care: clean cord care; immediate breastfeeding; thermal care.  INDIRECT: detect newborn complications and refer appropriately. Exclusive breastfeeding | INDIRECT: Increased ANC, facility delivery, clean delivery, identify danger signs, and appropriate referral. |

| **Table A3. continued. Characterisation Questions 4 and 5. Society for Family Health, 2014 update.** | | | | | |
| --- | --- | --- | --- | --- | --- |
| **Contact between the community volunteer and service users** | | | | | |
| Contact between frontline worker and service user -> | **Community volunteer and pregnant women** | **Community volunteer and women in labour** | **Community volunteer and new Mothers** | **Community volunteer and newborns** | **Community volunteer and husbands/ families** |
| **Routine contact ->** | New cadre for MNH, no established contact. Opportunistic contact | New cadre for MNH, no established contact. | New cadre for MNH, no established contact. | New cadre for MNH, no established contact. | New cadre for MNH, no established contact. Opportunistic contact only. |
| **Innovations enhancing this contact->** | 1. Mapping of service users and provision; 3. Train and deploy community volunteers; 4. FLW toolkit; 7. Mass media event. | 1. Mapping of service users and provision; 3. Train and deploy community volunteers 5. Call Centre; 6. Emergency Transport Scheme; 7. Mass media event; | 1. Mapping of service users and provision; 3.Train and deploy community volunteer 4. FLW toolkit; 5. Call Centre; 6. Emergency Transport Scheme; 7. Mass media event; | 1. Mapping of service users and provision; 3. Train and deploy community volunteers 4. FLW toolkit; 5. Call Centre; 6. Emergency Transport Scheme; 7. Mass media event; | 1. Mapping of service users and provision; 3. Train and deploy community volunteers 4. FLW toolkit; 5. Call Centre; 6. Emergency Transport Scheme; 7. Mass media event; |
| **Expected change in practice ->** | Complete and timely identification of pregnant women and registration for ANC. Effective delivery of key messages. Effective detection of danger signs and appropriate referral. | Complete and timely referral to appropriate delivery facility. Effective detection of danger signs and appropriate referral. | Complete and timely postpartum visit. Detection of danger signs, timely referral to appropriate facility if needed. | Complete and timely postnatal visit. Detection of danger signs, timely referral to appropriate facility if needed. | Engagement of husbands and families in care at all stages |
| **Expected effect on coverage of life-saving interventions (Direct or Indirect) ->** | INDIRECT: ID pregnancy; home visit, promote ANC; identify danger signs and appropriate referral. TT vaccine; Iron supplementation; malaria prevention; prevention and management of STIs including syphilis and HIV | INDIRECT: Promote facility delivery; family has access to clean delivery kit; identify danger signs and appropriate referral. For facility delivery prophylactic uterotonics to prevent PPH, management of PPH, AMTSL, access to C-sections. For home delivery: clean delivery including hand washing with soap and use of gloves by delivery attendant | DIRECT: Detect and treat maternal anaemia.  INDIRECT: Postpartum visit, ID danger signs and appropriate referral.  Detect danger signs e.g. post-partum sepsis and refer appropriately | Postnatal visit, ID danger signs and appropriate referral.  Home-based postnatal care so clean cord care; immediate and exclusive breastfeeding; thermal care; detection of newborn complications and appropriate referral | Promote ANC, facility delivery, postnatal care of mother and NB. |

| **Table A3. continued. Characterisation Questions 4 and 5. Society for Family Health, 2014 update.** | | | | | |
| --- | --- | --- | --- | --- | --- |
| **Contact between Call Centre staff and service users** | | | | | |
|  | **Call Centre staff and pregnant women** | **Call Centre staff and women in labour** | **Call Centre staff and new Mothers** | **Call Centre staff and newborns** | **Call Centre staff and husbands/ families** |
| **Routine contact ->** | New cadre, no established contact. | New cadre, no established contact. | New cadre, no established contact. | New cadre, no established contact. | New cadre, no established contact. |
| **Innovations enhancing this contact->** | 1. Mapping of service users and provision; 2. Train and deploy TBA; 3. Train and deploy community volunteer; 5. Call Centre; 6. Emergency Transport Scheme; 7. Mass media event. | 1. Mapping of service users and provision; 2. Train and deploy TBA; 3. Train and deploy community volunteer; 5. Call Centre; 6. Emergency Transport Scheme; 7. Mass media event. | 1. Mapping of service users and provision; 2. Train and deploy TBA; 3. Train and deploy community volunteer; 5. Call Centre; 6. Emergency Transport Scheme; 7. Mass media event. | 1. Mapping of service users and provision; 2. Train and deploy TBA; 3. Train and deploy community volunteer; 5. Call Centre; 6. Emergency Transport Scheme; 7. Mass media event. | 1. Mapping of service users and provision; 2. Train and deploy TBA; 3. Train and deploy community volunteer; 5. Call Centre; 6. Emergency Transport Scheme; 7. Mass media event. |
| **Expected change in practice ->** | Effective delivery of advice on danger signs, uptake of professional care, appropriate response to danger signs in pregnancy | Effective delivery of advice on danger signs, uptake of professional care, appropriate response to danger signs in labour | Effective delivery of advice on danger signs, uptake of professional care, appropriate response to postpartum danger signs | Effective delivery of advice on danger signs, uptake of professional care, appropriate response to postnatal danger signs | Effective delivery of advice on danger signs, uptake of professional care, appropriate response to danger signs at all stages |
| **Expected effect on coverage of life-saving interventions (Direct or Indirect) ->** | Information, advice and promotion of ANC so INDIRECTLY TT vaccine, iron supplementation, prevention of malaria, prevention and management of STIs including syphilis and HIV | Advise families to identify danger signs and appropriate referral  INDIRECTLY all intrapartum critical interventions | Advise families to identify danger signs and appropriate referral | Information, advice and promotion of postnatal care of newborn so indirect danger signs and refer appropriately  INDIRECTLY Clean cord care, thermal care; detection and appropriate management of complications | Information, advice and promotion of ANC, facility delivery, identify danger signs, postnatal care of mother and newborn so  INDIRECTLY, all critical interventions |

| **Table A3. continued. Characterisation Questions 4 and 5. Society for Family Health, 2014 update.** | | | | | |
| --- | --- | --- | --- | --- | --- |
| **Contact between Patent Medicine Vendors and service users** | | | | | |
|  | **Patent medicine vendors and pregnant women** | **Patent medicine vendors and women in labour** | **Patent medicine vendors and new mothers** | **Patent medicine vendors and newborns** | **Patent medicine vendors and husbands/ families** |
| **Routine contact ->** | Pregnant women buy standard cost Clean Delivery Kits | No routine contact. | No routine contact. | No routine contact. | No routine contact. |
| **Innovations enhancing this contact->** | 8. Mass media event; 9. Enhanced clean delivery kit supplies |  |  |  |  |
| **Expected change in practice ->** | Low cost clean delivery kits available |  |  |  |  |
| **Expected effect on coverage of life-saving interventions (Direct or Indirect) ->** |  | INDIRECT: Hand washing with soap and use of gloves by delivery attendant. |  | INDIRECT: Clean cord care. |  |

1. National Union of Road Transport Workers. ^2^ Frontline workers [↑](#footnote-ref-1)
